# Perioperative intravenous lidocaine for postoperative pain in patients undergoing breast surgery: a meta-analysis with trial sequential analysis of randomized controlled trials

**DOI:** 10.3389/fonc.2023.1101582

**Published:** 2023-06-23

**Authors:** Jia Li, Jiao Huang, Jiang-tao Yang, Jing-chen Liu

**Affiliations:** ^1^ Department of Anesthesiology, The First Affiliated Hospital, Guangxi Medical University, Nanning, China; ^2^ Department of Orthopedics, Guangxi Traditional Chinese Medical University Affiliated First Hospital, Nanning, China

**Keywords:** lidocaine, breast surgery, chronic post-surgical pain, opioid, meta-analysis

## Abstract

**Background:**

The effectiveness of intravenous lidocaine infusion in managing acute and chronic pain following breast surgery has been a topic of debate. This meta-analysis aims to assess the impact of perioperative intravenous lidocaine on the relief of postoperative pain among patients undergoing breast surgery.

**Methods:**

A systematic search of databases was conducted to identify randomized controlled trials (RCTs) that compared the effects of intravenous lidocaine infusion with placebo or routine care in patients undergoing breast surgery. The primary outcome of interest was the occurrence of chronic post-surgical pain (CPSP) at the longest follow-up. Meta-analyses, incorporating trial sequential analysis, were performed using a random-effects model to assess the overall effect.

**Results:**

A total of twelve trials, involving 879 patients, were included in the analysis. Perioperative intravenous lidocaine demonstrated a significant reduction in the incidence of CPSP at the longest follow-up (risk ratio [RR] 0.62, 95% confidence interval [CI] 0.48-0.81; P = 0.0005; I2 = 6%). Trial sequential analysis (TSA) indicated that the cumulative z curve crossed the trial sequential monitoring boundary for benefit, providing sufficient and conclusive evidence. Furthermore, intravenous lidocaine was associated with decreased opioid consumption and a shorter length of hospital stay.

**Conclusion:**

Perioperative intravenous lidocaine is effective in relieving acute and CPSP in patients undergoing breast surgery.

**Systematic review registration:**

https://inplasy.com/, identifier INPLASY2022100033.

## Introduction

Breast surgery is a widely performed procedure worldwide, with a significant number of patients experiencing moderate to severe acute pain (30-50%) (1, 2)and developing chronic post-surgical pain (CPSP) (25-68%) (3, 4).CPSP, characterized by persistent or worsening pain in the breast region lasting for at least 3 months after surgery (5, 6), can have detrimental effects on emotional well-being, functional abilities, quality of life, and impose substantial financial burdens on healthcare systems (4, 7). The pathophysiology of CPSP involves mechanisms such as traumatic nerve injury, neuroinflammation, and central neuronal sensitization (8). The conventional approach to managing postoperative pain relies heavily on opioids, which carries the risk of adverse effects including respiratory depression, addiction, and even mortality (9). To address these challenges, multimodal analgesic strategies have been proposed to alleviate both acute and chronic postoperative pain following breast surgery (10).

Lidocaine, being used originally as an antiarrhythmic agent, has been found to possess antinociceptive (11), anti-inflammatory (12) and anti-hyperalgesia (13) properties, making it a potentially useful drug for relieving postoperative pain. The systemic administration of lidocaine has shown efficacy in relieving neuropathic pain (14). Previous meta-analyses have demonstrated the effectiveness of intravenous lidocaine in reducing postoperative pain and opioid consumption in patients undergoing spine (15) and abdomen surgery (16, 17). However, the efficacy of intravenous lidocaine specifically for breast surgery has not been extensively evaluated due to limitations such as small sample sizes and conflicting findings from individual studies (18–22). Therefore, we conducted this meta-analysis to assess the efficacy of intravenous lidocaine in breast surgery patients. Our hypothesis was that perioperative intravenous lidocaine could alleviate both acute postoperative pain and chronic persistent post-surgical pain (CPSP) following breast surgery.

## Methods

This meta-analysis was performed in accordance with the Cochrane Handbook for Systematic Reviews of Interventions (23) and is reported in compliance with the updated PRISMA 2020 statement guideline (24). The protocol was registered on International Platform of Registered Systematic Review and Meta-analysis Protocols (INPLASY2022100033) (https://inplasy.com/).

### Literature search

A systematic electronic search was conducted in PubMed, Embase, and the Cochrane Library from their inception until September 20, 2022. The search strategy employed in PubMed was as follows: (lidocaine OR lignocaine OR xylocitin OR xylocaine OR lidocainum) AND (breast OR mastectomy OR mammaplasty). No restrictions were applied during the search process. In addition, the reference lists of the retrieved studies and previous reviews were examined to identify any additional potentially eligible trials for inclusion in the analysis.

### Study selection

The initial records were imported into EndNote software (Clarivate Analytics), and duplicate records were removed. Two authors (JL and JH) independently reviewed the titles and abstracts of the records to determine their relevance. The records were categorized as included, excluded, or requiring further evaluation. In cases where there was uncertainty, the full-text articles were obtained for further assessment of eligibility. Any disagreements regarding the inclusion of a trial were resolved through discussion between the authors.

### Eligibility criteria

The inclusion criteria for studies in this meta-analysis were as follows: (1) the study population consisted of adult patients undergoing breast surgery; (2) the intervention involved perioperative intravenous lidocaine; (3) a comparison group receiving either a control intervention or placebo was present; and (4) the study design was a randomized controlled trial (RCT). The primary outcome of interest was the occurrence of chronic post-surgical pain (CPSP) at the longest follow-up. Secondary outcomes included acute postoperative pain, morphine consumption during and after surgery, administration of postoperative rescue analgesics, postoperative nausea and vomiting (PONV), quality of recovery, and length of hospital stay.

### Data extraction

Data extraction was performed by JH and confirmed independently by other authors (JL and JTY). We used a predefined data extraction form (Excel, Microsoft Corporation, USA) to collect the following information: first author, year of publication, country, population, ASA classification, surgical procedure, number of patients, intervention (route, dosage, and duration of lidocaine), comparison, and outcomes.

### Risk of bias

Two authors (JL and JH) assessed risk of bias independently, using the Cochrane risk-of-bias tool (25) including random sequence generation (selection bias), allocation concealment (selection bias), blinding of participants and personnel (performance bias), blinding of outcome assessment (detection bias), incomplete outcome data (attrition bias), selective reporting (reporting bias), and other bias. Each item was rated as low, unclear, or high risk of bias. Trials with ≥1 key domains at high risk of bias were considered as at high risk of bias; trials with all key domains at low risk of bias were classified into low risk of bias; otherwise, they were considered to be at unclear risk of bias.

### Grading quality of evidence

The certainty of evidence was evaluated using the Grading of Recommendations Assessment, Development, and Evaluation (GRADE) (26) system. The assessment considered factors such as risk of bias, inconsistency, indirectness, imprecision, and publication bias. Based on these criteria, the quality of evidence was categorized as very low, low, moderate, or high. The GRADE Profiler (version 3.6, GRADE pro) was utilized to construct a summary table presenting the findings.

### Statistical analysis

Summary statistics were reported as relative risks (RRs) with 95% confidence intervals (CIs) for dichotomous outcomes and mean differences (MDs) with 95% CIs for continuous outcomes. Pooled data were analyzed using random-effects models based on the intention-to-treat principle. Heterogeneity across the trials were evaluated by Cochrane Q test (P < 0.1) and the quantitative I^2^ statistic (I^2^ >50%) (27, 28). Regardless of heterogeneity, outcome data were synthesized using a random-effects model. Subgroup analyses of CPSP were conducted based on the duration of follow-up. Publication bias was assessed visually using a funnel plot and also evaluated using Begg’s and Egger’s tests (29, 30). Statistical significance was considered at a two-sided P-value less than 0.05, unless otherwise specified. All statistical analyses were performed using RevMan 5.4 (Nordic Cochrane Centre) and Stata version 12.0 (Stata Corp LP).

### Trial sequential analysis

Interim analyses in a single trial can increase the risk of type I error (false-positive results). To avoid this issue, monitoring boundaries can be implemented to determine whether a trial should be terminated early based on a sufficiently small P-value indicating the anticipated effect or futility. Similarly, meta-analysis with small sample sizes may increase the type I error results due to the sparse data and repetitive testing of accumulating data (31). Trial sequential analysis (TSA) is a new statistical method to addressing these challenges. It can generate the monitoring boundaries, required information size, and futility boundaries to determine whether the evidence in a meta-analysis is reliable and conclusive. If the cumulative Z curve reaches the required information size (RIS) line or enters the trial sequential monitoring boundary, it indicates that sufficient evidence to achieve the anticipated effect of intervention and no further trials are need. If the Z curve does not cross any of the boundaries and has not reach the RIS, there is insufficient evidence to draw conclusion. We used TSA to estimate the RIS for this meta-analysis. Parameters for calculating RIS include type I error (α = 0.05, two-sided), type II error (β= 0.20, power of 80%), the control event proportions, and the RR reduction of 20% for primary outcomes. Trial Sequential Analysis Viewer version 0.9 Beta was used for these analyses (32).

## Results

### Trial selection

**Figure 1:**
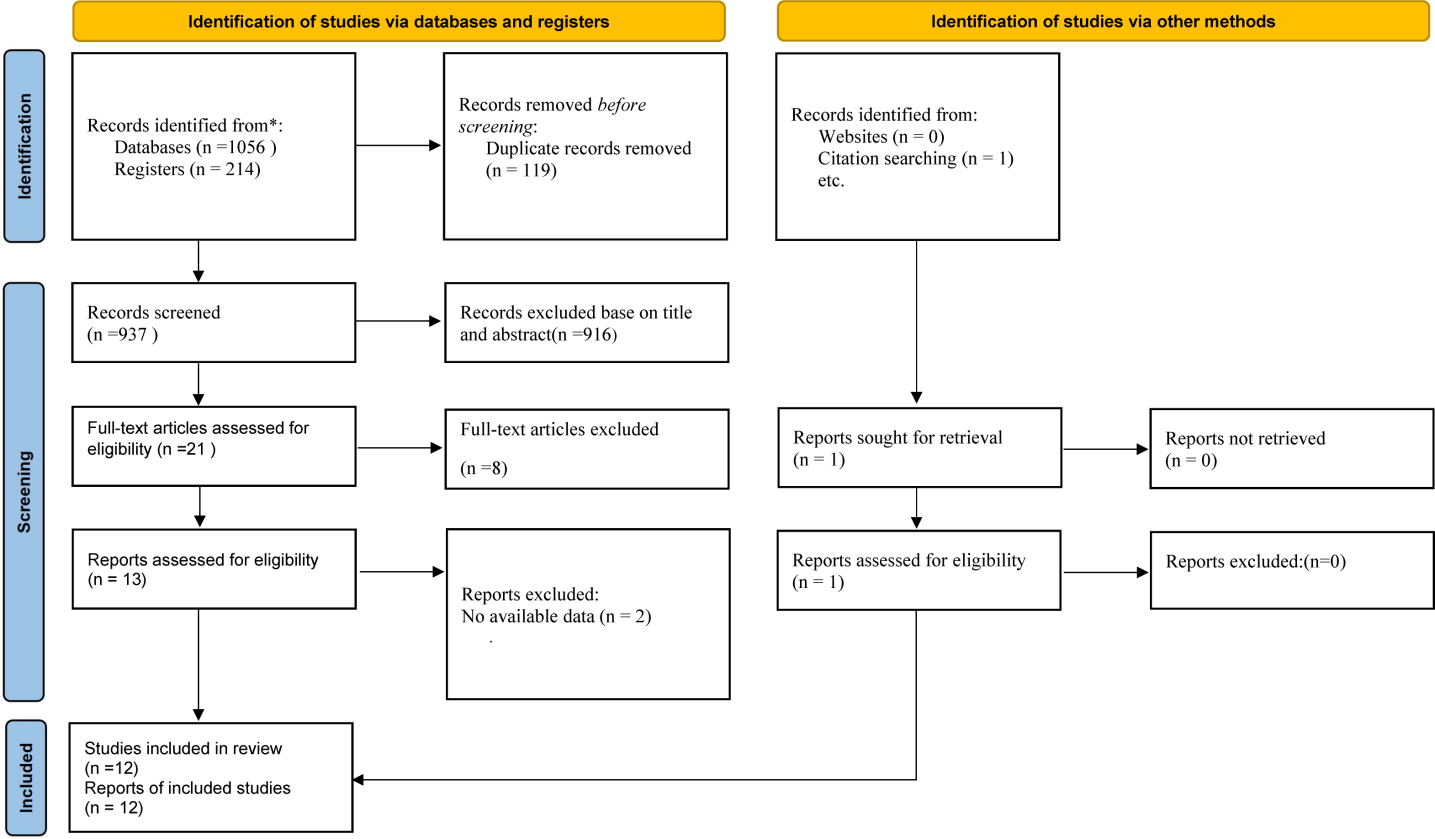
Preferred Reporting Items for Systematic Reviews and Meta-Analyses (PRISMA) diagram summarizing literature screening process.

The initial search resulted in a total of 1270 records, out of which 119 duplicates were removed. Following the screening of titles and abstracts, the full texts of the remaining 21 articles were assessed for eligibility. Ultimately, twelve randomized controlled trials (RCTs) met the inclusion criteria and were included in the meta-analysis (6, 18–22, 33–38) (**Figure 1**).

### Trial characteristics

The main characteristics of the included trials are summarized in **Table 1**. These trials were published between 2012 and 2022. The sample size ranged from 37 to 150, with a total of 879 participants. Among the included trials, three trials were multi-center (22, 35, 37), the remaining nine were single-center (6, 18–21, 33, 34, 36, 38). All trials except Choi et al. (33) recruited patients undergoing breast cancer surgery. All trials’ route of administration of lidocaine are intravenous injection except Toner et al. (37) used postoperative subcutaneous lidocaine for 12 hours after completion of surgery. The dosage of continuous lidocaine infusion ranges from 1.5mg/kg/h to 2mg/kg/h throughout the breast surgery. The continuous lidocaine infusion was stopped at 1 hour after the start of surgery (20), at the starting (22) or end of skin closure (33), 1 hour after the surgical closure (18, 35), before transferring patients into the recovery room (21), 2 hours after arrival in the recovery room (19, 34), the end of surgery (6, 38), 1 hour (36) and 12 hours after surgery (37).

**Table 1:** Study Characteristics.

Study	Design	Population	ASA	Surgical procedure	No. of patients	Intervention	Lidocaine stopping time	Comparison	Outcomes
Choi 2012	Single-centre in Korea	Adults females between 20-60	I-II	Elective plastic breast surgeries	60	Lidocaine 1.5 mg/kg i.v. 30 min before incision, followed by continuous infusion 1.5 mg/kg/h throughout the surgery	The end of skin closure	No infusion	Pain score, rescue analgesic administration PONV, hospital stay
Grigoras 2012	Single-centre in Ireland	Adults females	I-II	Breast cancer surgeries	37	Lidocaine 1.5 mg/kg i.v. 10 min after inctubation followed by a continuous infusion at 1.5 mg/kg/h throughout the surgery	1 hour after the surgical closure	Saline	Intraoperative and postoperative opioid consumption, PONV, CPSP
Terkawi 2014, 2015	Signal-centers in America	Adults females between 18-80	I–III	Breast cancer surgeries	80	Lidocaine 1.5 mg/kg i.v. after anesthetic induction followed by a continuous infusion at 2.0 mg/kg/h throughout the surgery	2 hours after arrival in the recovery room	Saline	Intraoperative and postoperative opioid consumption, pain score, PONV, CPSP, hospital stay
Couceiro 2015	Single-centre in Brazil	Adults females between 18-75	NA	Breast cancer surgery	44	Lidocaine 3 mg/kg i.v. 60 min after incision	1 hour after surgery starting	Saline	Rescue analgesic administration
Kim 2017	Single-centre in Korea	Adults females between 20-65	I-II	Breast cancer surgery	84	Lidocaine 2mg/kg i.v. after anesthetic induction, followed by continuous infusion 2 mg/kg/h throughout the surgery	Before transferring patients to the recovery room	Saline	Intraoperative and postoperative opioid consumption, pain score, rescue analgesics administration, quality of recovery, CPSP
Kendall 2018	Multicentre in America	Adults females between 18-70	I–III	Breast cancer surgery	150	Lidocaine 1.5mg/kg i.v. after anesthetic induction, followed by continuous infusion 2 mg/kg/h throughout the surgery	Discontinued one hour following placement of the last suture	Saline	Intraoperative and postoperative opioid consumption, quality of recovery, CPSP
Khan 2019	Multicentre in Canada	Adults females between 18-75	NA	Breast cancer surgery or mastectomy	100	Lidocaine 1.5mg/kg i.v.after anesthetic induction, followed by continuous infusion 2 mg/kg/h throughout the surgery	The start of surgical closure	Lidocaine placebo	Intraoperative opioid consumption, PONV, CPSP
Van den Heuve 2020	Single-centre in Netherlands	Adults females	NA	Breast cancer surgery	30	lidocaine of 1.5 mg/kg i.v. 10 min before anesthetic induction, followed by continuous infusion 2 mg/kg/h throughout the surgery	1 hour after end of surgery	Saline	Pain score
Toner 2021	Multicentre in Australian	Adults females between 18-80	I–III	Breast cancer surgery	150	Lidocaine 1.5mg/kg i.v. over 5min after anesthetic induction, followed by continuous infusion 2 mg/kg/h throughout the surgery	12 hours after surgery via the subcutaneous route	Saline	Pain score, PONV, rescue analgesics administration, quality of recovery, CPSP, hospital stay
Wei 2022	Single-centre in China	Adults females between 18-85	I–III	Breast cancer surgery	62	Lidocaine 1.5mg/kg i.v. 10min before anesthetic induction, followed by continuous infusion 2 mg/kg/h throughout the surgery	The end of surgery	Saline	Intraoperative opioid consumption, PONV, rescue analgesics administration, quality of recovery,
Xia 2022	Single-centre in China	Adults females between 18-85	I–III	Breast cancer surgery	82	Lidocaine 1.5mg/kg i.v. 10min before anesthetic induction, followed by continuous infusion 2 mg/kg/h throughout the surgery	The end of surgery	Saline	Pain score and rescue analgesic administration, CPSP

ASA, American Society of Anesthesiology.

### Risk of bias assessment

**Figure 2:**
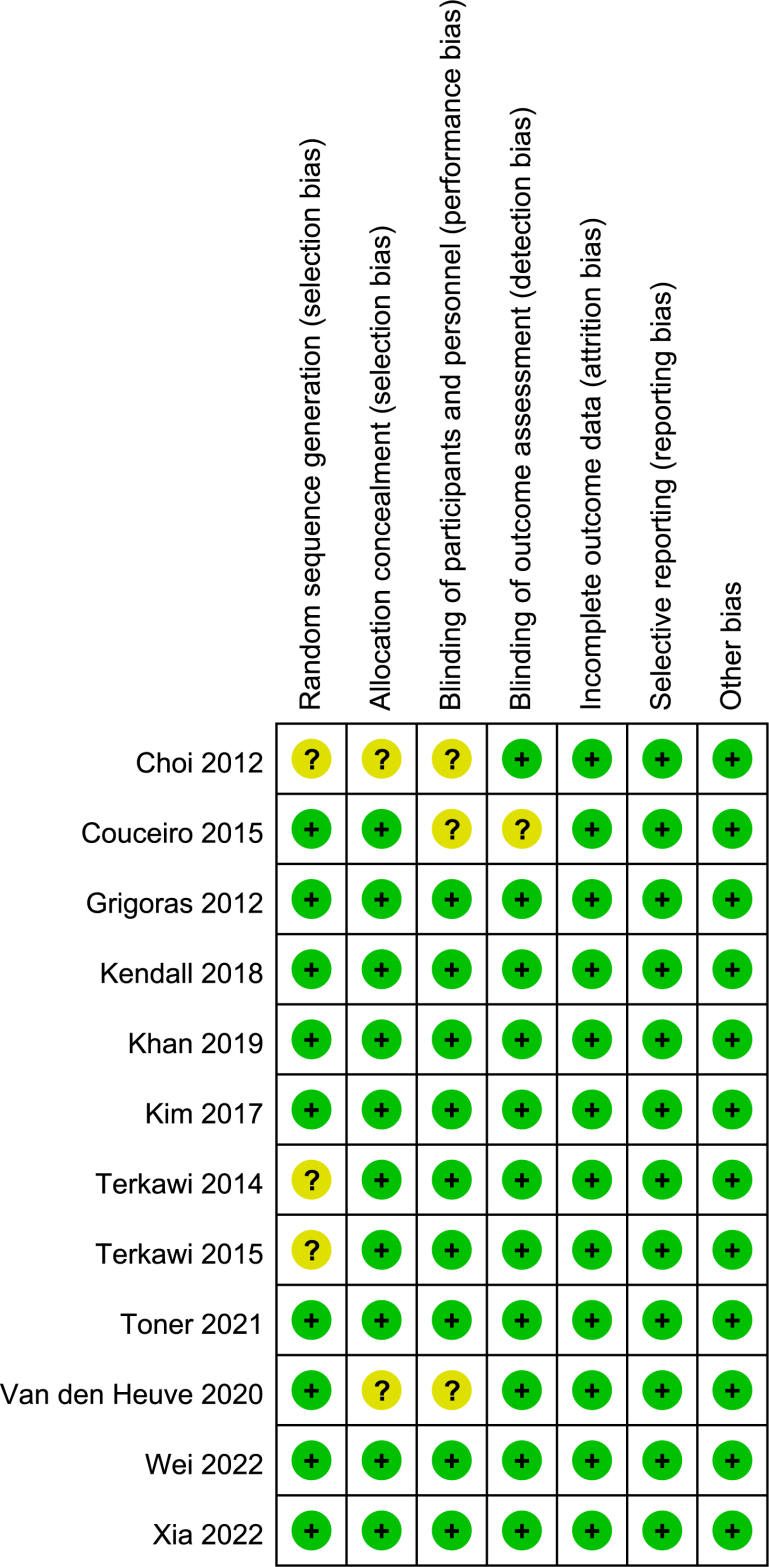
Risk of bias summary.

Details of risks of bias across the included trials are presented in **Figure 2**. Out of the twelve trials, seven were classified as having a low risk of bias (6, 18, 21, 22, 35, 37, 38), while the remaining five trials were categorized as being at unclear risk of bias (19, 20, 33, 34, 36). Nine trials reported the generation of an adequate randomized sequence (6, 18, 20–22, 35–38), and three trials provided information on appropriate allocation concealment (20, 33, 36).

### Chronic post-surgical pain

**Figure 3:**
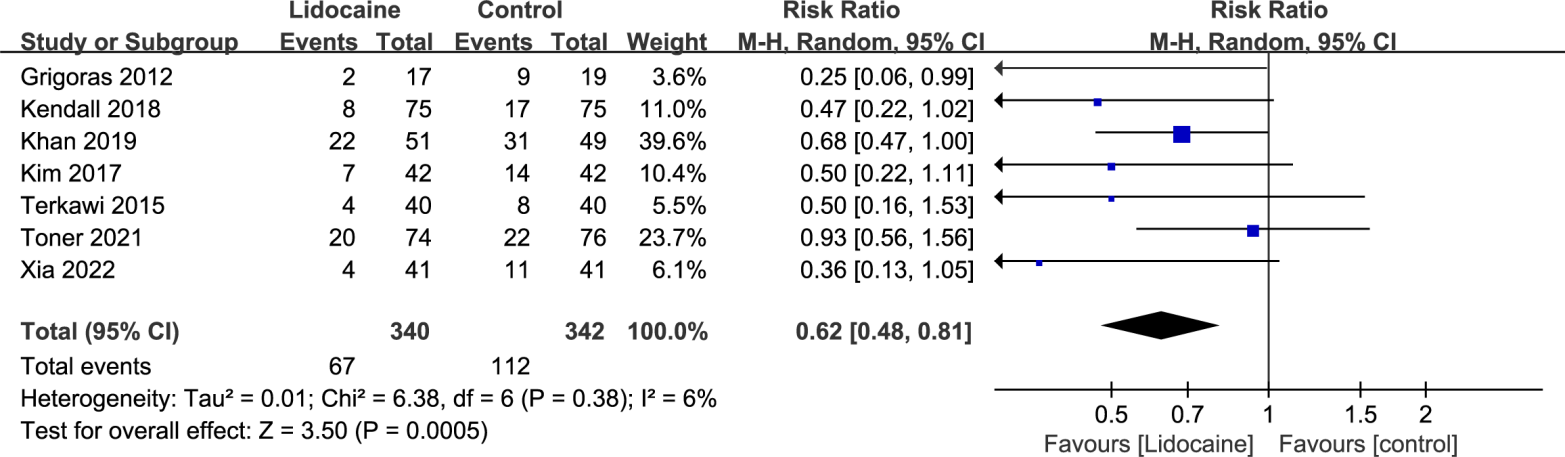
Forest plot for chronic post-surgical pain.

**Figure 4:**
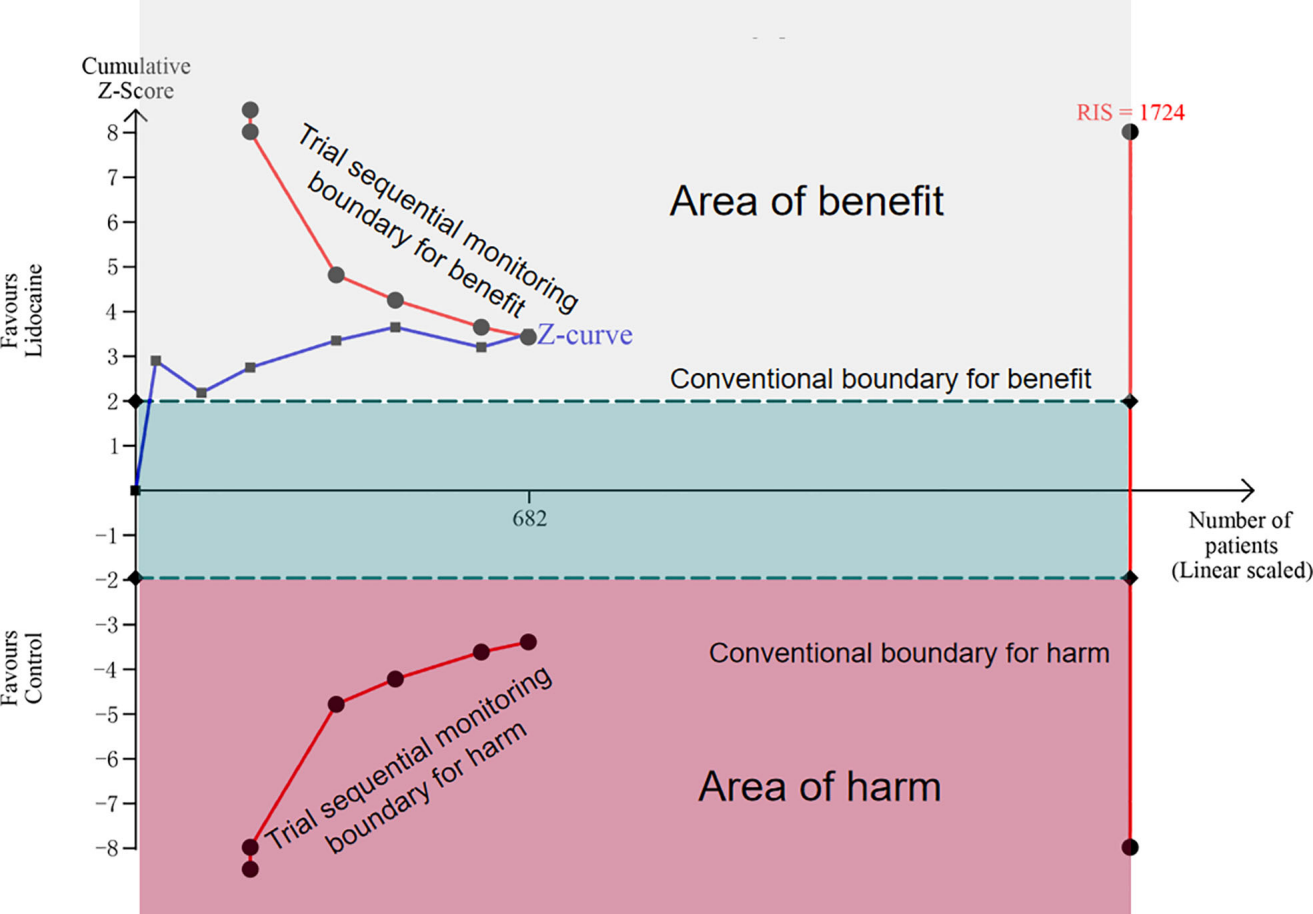
Trial sequential analysis for chronic post-surgical pain at the longest follow up (scaled trial distance). Trial sequential analysis of seven trials (black filled squares) illustrating that the cumulative Z curve crossed the conventional boundary and the trial sequential monitoring boundary for benefit, establishing sufficient and conclusive evidence. A diversity-adjusted required information size of 1724 patients were calculated using α = 0.05 (two-sided), β= 0.20 (power of 80%), an anticipated relative risk reduction of 20%, and an event proportion of 32.75% in the control group.

Seven trials included in this meta-analysis provided data on CPSP at the longest follow-up. Pooled analysis suggested that intravenous lidocaine significantly reduced the incidence of CPSP at the longest follow-up (seven trials; RR 0.62, 95% CI 0.48-0.81; P = 0.0005; **Figure 3**; **Table 2**). There was no significant heterogeneity observed across the studies (I^2 ^= 6%). These findings remained consistent when subgroup analyses were performed based on the follow-up time of CPSP (**Supplementary Material Figure S1**). The trial sequential analysis (TSA) indicated that the cumulative Z curve crossed both the conventional boundary and the trial sequential monitoring boundary for benefit, establishing sufficient and conclusive evidence (**Figure 4** and **Supplementary Material Figure S2**).

**Table 2:** The outcomes of analysis.

Outcomes	No of patients	No of studies	Effect SMD/ RR(95%CI)	P	I^2^
CPSP	682	7	RR 0.62(0.48-0.81)	0.0005	6%
Pain scores at movement at 2h	160	2	SMD -0.63(-0.91 to -0.35)	0.00001	0%
Pain scores at rest at 2h	198	3	SMD -0.73; ( -1.00, -0.46)	0.00001	12%
Pain scores at rest at 4h	138	3	SMD -1.03( -1.40 ,-0.65)	0.00001	0%
Pain scores at rest at 48h	276	4	SMD -0.45(-0.67, -0.23)	0.0001	0%
Pain scores at rest at 72h	196	3	SMD -0.59(-0.99,-0.20)	0.003	29%
Remifentanil consumption	376	4	SMD -187.40(-238.37, -136.44)	0.00001	0%
Morphine consumption at 24h after breast surgery	450	5	SMD -0.78( -1.04 to -0.52)	0.00001	0%

CPSP, chronic post-surgical pain; SMD, standard mean difference; RR, risk ratio; CI, confidence interval.

### Acute postoperative pain scores

**Figure 5:**
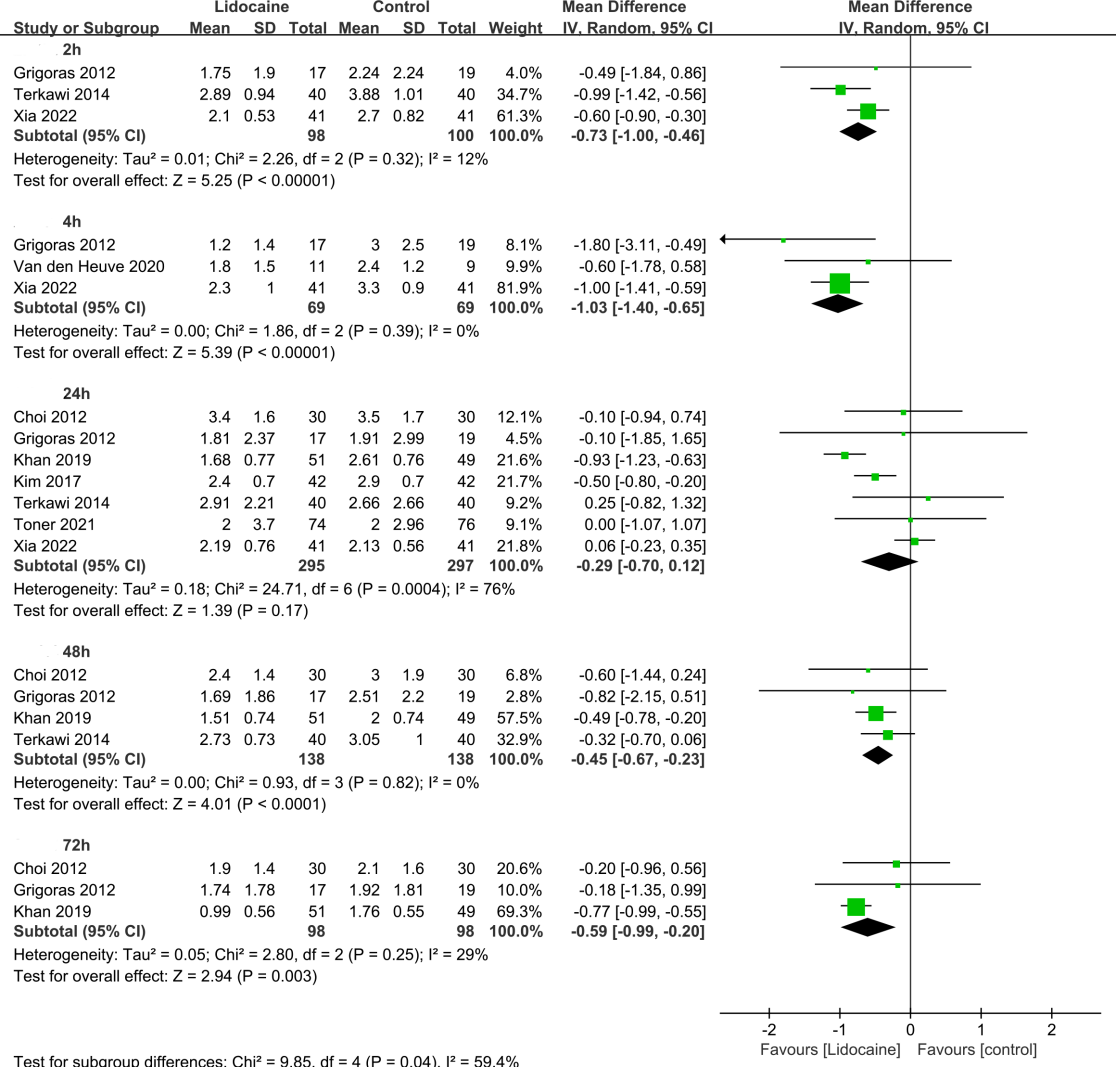
Forest plot for acute post-surgical pain at rest.

**Figure 6:**
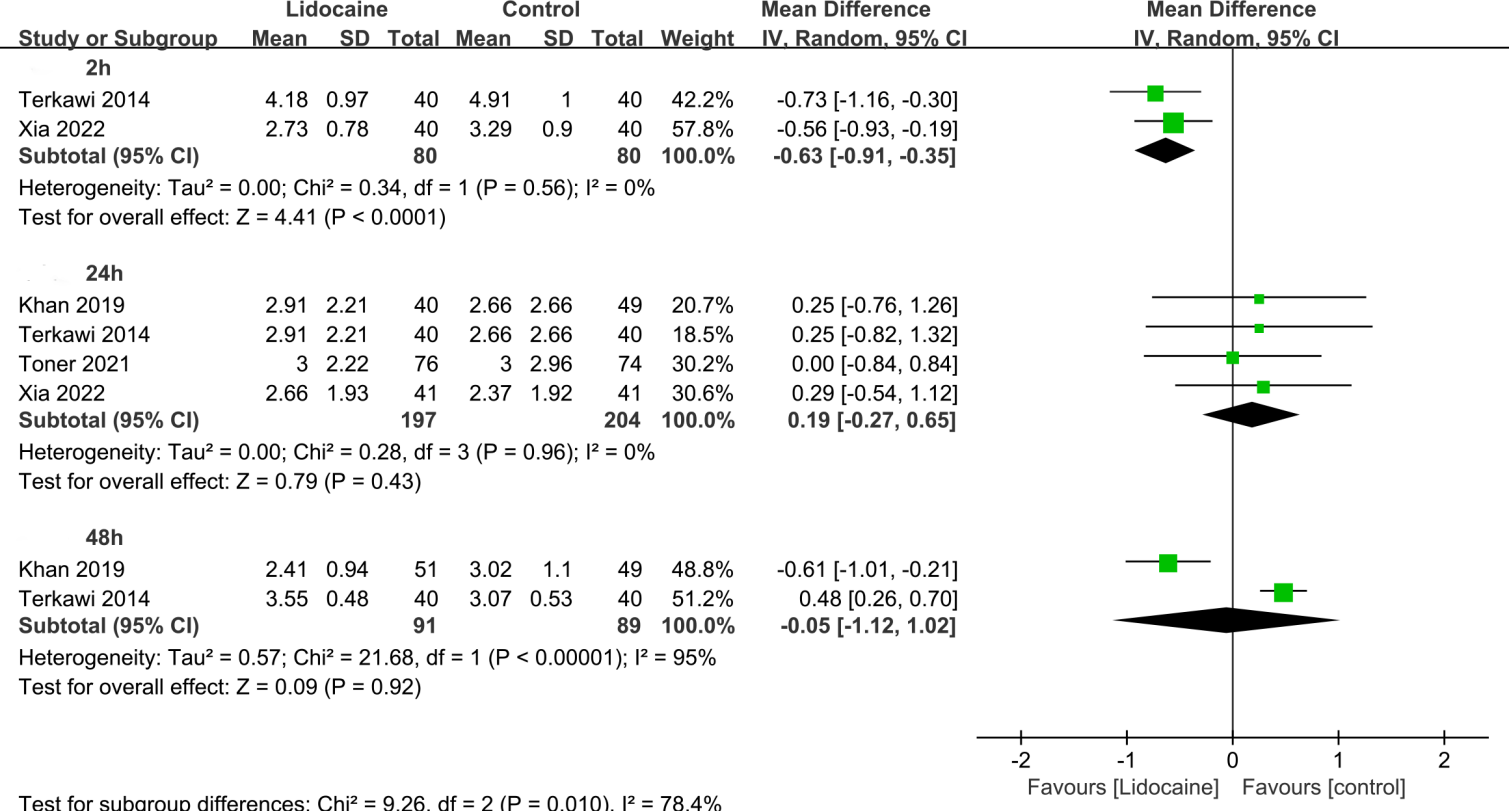
Forest plot for acute post-surgical pain at movement.

This meta-analysis examined the postoperative pain scores assessed using Visual Analogue Scale or Numerical Rating Scale at rest and during movement (**Table 2**). The pooled analysis demonstrated that postoperative pain scores at rest were significantly lower in the lidocaine group compared to the control group at 2h (three trials; MD -0.73; 95% CI -1.00 to -0.46; P < 0.00001; I^2 ^= 12%), 4h (three trials; MD -1.03; 95% CI -1.40 to -0.65; P < 0.00001; I^2 ^= 0%), 48h (four trials; MD -0.45; 95% CI -0.67 to -0.23; P < 0.0001; I^2 ^= 0%), and 72h (three trials; MD -0.59; 95% CI -0.99 to -0.20; P < 0.003; I^2^= 29%; **Figure 5**). Similarly, postoperative pain scores during movement were significantly lower in the lidocaine group at 2h (MD -0.63; 95% CI -0.91 to -0.35; P < 0.00001; **Figure 6**). Although no statistically significant difference was observed in pain scores between the two groups at 24 hours after surgery, there was a trend towards improved pain control in the intravenous lidocaine group.

### Opioid consumption

The administration of perioperative intravenous lidocaine was found to be associated with a significant reduction in remifentanil consumption during surgery (MD -187.40; 95% CI -238.37 to -136.44; P < 0.00001; **Table 2**) without significant heterogeneity (I^2 ^= 0%; **Supplementary Material Figure S3**). Additionally, it was observed that lidocaine resulted in decreased morphine consumption at 24 hours after breast surgery (MD -0.78; 95% CI -1.04 to -0.52; P < 0.00001; **Table 2**), with no significant heterogeneity (I^2 ^= 0%; **Supplementary Material Figure S4**). However, there were no significant differences in morphine consumption during surgery between the lidocaine and placebo groups (**Supplementary Material Figure S5**).

### Recovery indices

The pooled estimates revealed that there was no significant difference in the incidence of postoperative nausea or vomiting (PONV) between the lidocaine and placebo groups (RR 0.95, 95% CI 0.69-1.31; P = 0.75; **Supplementary Material Figure S6**). Similarly, there was no significant difference in the administration of rescue analgesics within 24 hours after surgery between the lidocaine and placebo groups (RR 0.95, 95% CI 0.84-1.06; P = 0.33; **Supplementary Material Figure S7**). However, the length of hospital stay was significantly shorter in the lidocaine group (MD -1.11; 95% CI -2.12 to -0.1; P < 0.003; I2 = 0%; **Supplementary Material Figure S8**). Furthermore, the quality of postoperative recovery within 24 hours after surgery was comparable between the lidocaine and placebo groups (**Supplementary Material Figure S9**).

### Adverse events

Four trials reported intravenous lidocaine associated side effects (19, 22, 37, 38). No toxicity cases were found in trials performed by Terkawi et al. (19), Khan et al. (22) and Wei et al. (38). However, Toner et al. (37) specifically monitored the occurrence of toxicity for 12 hours and reported that three patients in the lidocaine group and one patient in the control group experienced a metallic taste. Due to the limited amount of data available, pooled analyses were not conducted for these side effects.

### Certainty of evidence

The GRADE evidence profiles for the outcomes are presented in **Supplementary Material Table S1**. The level of evidence according to GRADE is classified as moderate for chronic postoperative pain scores (CPSP), acute postoperative pain scores at most time points, and remifentanil consumption during surgery. For other outcomes, such as postoperative nausea and vomiting (PONV) and hospital stay, the level of evidence is categorized as low.

### Publication bias

Visual inspection suggested that the funnel plot for CPSP appear to be asymmetrical. But no publication bias was detected in formal statistical tests (Begg´s test, P = 0.764; Egger´s test, P = 0.401; **Supplementary Material Figure S10** **).**

## Discussion

### Principal findings

Our comprehensive and systematic meta-analysis examined the available literature and revealed several key findings. Firstly, perioperative intravenous lidocaine demonstrated a significant reduction in chronic postoperative pain (CPSP) following breast surgery. This beneficial effect was consistent across subgroup analyses and was further supported by trial sequential analysis (TSA). Secondly, perioperative intravenous lidocaine exhibited positive effects in alleviating acute pain after breast surgery. Thirdly, the use of perioperative intravenous lidocaine resulted in reduced opioid consumption during and 24 hours after surgery. Fourthly, patients receiving perioperative intravenous lidocaine experienced a shorter hospital stay compared to those who did not. Lastly, there was no observed benefit of perioperative intravenous lidocaine on the quality of recovery within 24 hours after surgery. These findings provide valuable insights into the potential benefits of perioperative intravenous lidocaine in breast surgery patients.

### Comparison with previous meta-analyses

In a previous meta-analysis conducted by Chang et al. (39), they explored the potential benefits of perioperative intravenous lidocaine in postoperative pain management. However, their study was limited by a small sample size, including only four randomized controlled trials (RCTs) involving 167 patients. Consequently, their findings lacked sufficient statistical power to draw conclusive results regarding the effects of intravenous lidocaine on acute pain after breast surgery. In contrast, our current meta-analysis overcomes this limitation by including a larger pool of evidence comprising 12 RCTs with a total of 879 patients. The increased statistical power provided by our larger sample size allows for more robust conclusions to be drawn. Our findings demonstrate significant benefits of perioperative intravenous lidocaine in reducing the incidence of chronic postoperative pain (CPSP). To ensure a conservative estimate, we employed trial sequential analysis (TSA), which further supported the sufficiency and conclusiveness of the evidence. Inconsistent with Chang et al. (39), our pooled analyses showed significant benefits of intravenous lidocaine on alleviating acute pain (2h, 4h, 48h, 72h at rest) after breast surgery. In addition, we found perioperative intravenous lidocaine was associated with reduced opioid consumption during and 24h after breast surgery. Lastly, we expanded our assessment to include various recovery indices such as the quality of postoperative recovery, postoperative nausea and vomiting (PONV), and hospital stay, in order to provide a comprehensive evaluation of the effects of intravenous lidocaine compared to the control group.

### Possible mechanisms for findings

The benefits of perioperative intravenous lidocaine in postoperative pain management can be attributed to three theoretical mechanisms: its analgesic (11), anti-inflammatory (12) and anti-hyperalgesia (13) properties, which have been previously described. The analgesic effect of lidocaine is primarily achieved through the blockade of voltage-gated sodium channels, resulting in a reversible inhibition of action potential propagation (40). Lidocaine also blocks potassium currents, which are important regulators of resting potential in neural transmission. By modulating these channels, lidocaine exerts its analgesic effects (11, 39). In terms of anti-inflammatory properties, neuro-inflammation plays a crucial role in the development of chronic postoperative pain. Lidocaine has been shown to down-regulate nuclear factor-kappa B and protein kinase C, leading to a decrease in neutrophil recruitment and a reduction in the release of pro-inflammatory cytokines such as IL-4 and IL-6 (41, 42). This anti-inflammatory action contributes to the attenuation of pain. Microglia, a type of immune cell in the central nervous system, are believed to be involved in nociceptive transmission (42). Lidocaine can directly act on microglia by inhibiting the increase of intracellular calcium (43), which may further contribute to its analgesic and anti-inflammatory effects. Furthermore, lidocaine exhibits anti-hyperalgesia properties by blocking N-methyl-D-aspartate (NMDA) receptors. NMDA receptors are particularly implicated in the transmission of pathological pain signals. By blocking these receptors, lidocaine helps reduce hyperalgesia, thereby providing additional pain relief (11, 44). These mechanisms collectively contribute to the overall efficacy of perioperative intravenous lidocaine in mitigating postoperative pain and its associated complications.

### Implications for clinical practice

Our findings have significant implications for clinical practice. Although our meta-analysis revealed positive effects of perioperative intravenous lidocaine in alleviating acute and chronic pain after breast surgery, caution should be exercised regarding the widespread use of intravenous lidocaine in clinical practice. One primary concern is the uncertain safety profile of intravenous lidocaine in managing postoperative pain. Among the trials included in this meta-analysis, only four reported adverse events associated with intravenous lidocaine. Due to the limited availability of data, we were unable to provide a comprehensive estimate of the adverse outcomes. Therefore, further high-quality, large-scale, prospective, multicenter trials are required to clarify the safety profile of intravenous lidocaine in reducing pain after breast surgery. Another important consideration is the narrow therapeutic window and potential toxicity of lidocaine (45), Clinicians should remain mindful of the possibility of lidocaine toxicity and strictly adhere to recommended doses and duration when using lidocaine. Recent guidelines on the management of intravenous lidocaine recommend an initial dose of no more than 1.5 mg/kg administered over a 10-minute period, calculated based on the patient’s ideal body weight. Considering the pharmacokinetic characteristics of lidocaine, even with continuous intravenous infusion at a dose of 3 mg/kg/hour, the resulting plasma concentration is 2.6 μg/ml (45), which remains below the toxic range of 8-12 μg/ml (46). There is data to support safe administration to 2 mg/kg/hour (47). The elimination half-life of lidocaine typically ranges from 90 to 120 minutes in most patients. However, this half-life may be prolonged in obese patients (48)or in patients with hepatic injury or congestive heart failure (11, 49). In such cases, there is a potential risk of lidocaine accumulation with continuous infusion, leading to intoxication. Therefore, it is crucial for clinicians to individualize lidocaine therapy based on the specific characteristics and needs of each patient.

## Strengths and limitation

This meta-analysis possesses a significant strength as it was registered in INPLASY and meticulously reported following the updated PRISMA guidelines. In order to enhance the robustness of our findings, we employed Trial Sequential Analysis (TSA) to assess the impact of intravenous lidocaine on chronic postsurgical pain (CPSP) following breast surgery. However, our meta-analysis does have certain limitations.

Firstly, although no substantial statistical heterogeneity was observed, the variation in patient ages across the included studies, as well as differences in anesthetic techniques, may influence the reliability of the results. Secondly, according to the GRADE system, the majority of evidence for both primary and secondary outcomes was determined to be of low to moderate quality.Lastly, due to limited data available from the included trials, we were unable to calculate the effect of the duration of intravenous lidocaine administration on CPSP and other outcomes.

## Conclusions

The existing evidence strongly indicates that perioperative intravenous lidocaine administration effectively reduces both acute and chronic pain following breast surgery.

## Data availability statement

The original contributions presented in the study are included in the article/**Supplementary Material**. Further inquiries can be directed to the corresponding author.

## Authors contributions

JL and JH: Study conception and design; acquisition of and analysis the data; drafting of the article; Revising it critically for important intellectual content. J-TY: Acquisition and analysis of data; statistical reviewer; revising the article. All authors contributed to the article and approved the submitted version.
