# Supplementary material for: Perioperative intravenous lidocaine for postoperative pain in patients undergoing breast surgery: a meta-analysis with trial sequential analysis of randomized controlled trials
Source: Front Oncol. 2023 Jun 23;13:1101582. doi: 10.3389/fonc.2023.1101582 (PMC10327428; doi:10.3389/fonc.2023.1101582)
Supplement: Supplementary file 1 [file DataSheet_1.pdf]

Supplementary Online Content

Table S1. GRADE profile

Table S2. PRISMA 2020 checklist

Figure S1 Forest plot for chronic post-surgical pain at three and six months follow-up after breast surgery

Figure S2 Trial sequential analysis for chronic post-surgical pain at the longest follow-up (scaled trial distance)

Figure S3 Forest plot for remifentanil (μg) consumption during breast surgery

Figure S4 Forest plot for morphine(mg) consumption at 24h after breast surgery

Figure S5 Forest plot for morphine consumption during surgery

Figure S6 Forest plot for PONV within 24h breast surgery

Figure S7 Forest plot showing rescue analgesic administration within 24h surgery

Figure S8 Forest plot for length of hospital stay (in hours) after surgery

Figure S9 Forest plot showing quality of postoperative recovery within 24h after surgery

Figure S10 Begg's test for evaluating publication bias. RR, relative risks

Table S1. GRADE profile

Certainty assessment							Summary of findings				
Participants (studies) Follow-up	Risk of bias	Inconsistency	Indirectness	Imprecision	Publication bias	Overall certainty of evidence	Study event rates (%)		Relative effect (95% CI)	Anticipated absolute effects	
							With placebo	With CPSP		Risk with placebo	Risk difference with CPSP
**CPSP**											
682 (7 RCTs)	serious[a]	not serious	not serious	not serious	none	⨁⨁⨁◯ Moderate	112/342 (32.7%)	67/340 (19.7%)	**RR 0.62** (0.48 to 0.81)	327 per 1,000	**124 fewer per 1,000** (from 170 fewer to 62 fewer)
**Pain score at rest - 2h**											
198 (3 RCTs)	serious[a]	not serious	not serious	not serious	none	⨁⨁⨁◯ Moderate	100	98	-	The mean pain score at rest - 2h was **0**	MD **0.73 lower** (1 lower to 0.46 lower)
**Pain score at rest - 4h**											
138 (3 RCTs)	serious[a]	not serious	not serious	not serious	none	⨁⨁⨁◯ Moderate	69	69	-	The mean pain score at rest - 4h was **0**	MD **1.03 lower** (1.4 lower to 0.65 lower)
**Pain score at rest -24h**											
592 (7 RCTs)	serious[a]	serious[b]	not serious	not serious	none	⨁⨁◯◯ Low	297	295	-	The mean pain score at rest -24h was **0**	MD **0.29 lower** (0.7 lower to 0.12 higher)
**Pain score at rest - 48h**											
276 (4 RCTs)	serious[a]	not serious	not serious	not serious	none	⨁⨁⨁◯ Moderate	138	138	-	The mean pain score at rest - 48h was **0**	MD **0.45 lower** (0.67 lower to 0.23 lower)
**Pain score at rest - 72h**											
196 (3 RCTs)	serious[a]	not serious	not serious	not serious	none	⨁⨁⨁◯ Moderate	98	98	-	The mean pain score at rest - 72h was **0**	MD **0.59 lower** (0.99 lower to 0.2 lower)

Table S1. GRADE profile

Certainty assessment							Summary of findings			
**Pain score at movement - 2h**										
162 (2 RCTs)	serious[a]	not serious	not serious	not serious	none	⨁⨁⨁◯ Moderate	81	81	-	The mean pain score at movement - 2h was **0**	MD **0.63 lower** (0.91 lower to 0.35 lower)
**Pain score at movement - 24h**										
412 (4 RCTs)	serious[a]	not serious	not serious	not serious	none	⨁⨁⨁◯ Moderate	208	204	-	The mean pain score at movement - 24h was **0**	MD **0.35 lower** (1.63 lower to 0.92 higher)
**Pain score at movement - 48h**										
180 (2 RCTs)	serious[a]	serious[b]	not serious	not serious	none	⨁⨁◯◯ Low	89	91	-	The mean pain score at movement - 48h was **0**	MD **0.05 lower** (1.12 lower to 1.02 higher)
**Remifentanil consumption during surgery**										
378 (4 RCTs)	not serious	not serious	not serious	serious[c]	none	⨁⨁⨁◯ Moderate	189	189	-	The mean remifentanil consumption during surgery was **0**	MD **187.4 lower** (238.37 lower to 136.44 lower)
**Morphine consumption during surgery**										
366 (4 RCTs)	serious[a]	serious[b]	not serious	not serious	none	⨁⨁◯◯ Low	184	182	-	The mean Morphine consumption during surgery was **0**	MD **0.66 lower** (3.14 lower to 1.82 higher)
**Morphine consumption at 24h after surgery**										
450 (5 RCTs)	serious[a]	not serious	not serious	serious[c]	none	⨁⨁◯◯ Low	225	225	-	The mean morphine consumption at 24h after surgery was	MD **0.78 lower** (1.04 lower to 0.52 lower)

Table S1. GRADE profile

Certainty assessment							Summary of findings				
										**0**	
**Rescue analgesic administration**											
519<br>(7 RCTs)	serious[a]	not serious	not serious	serious[c]	none	⊕⊕◯◯<br>Low	114/262<br>(43.5%)	108/257<br>(42.0%)	**RR 0.95**<br>(0.84 to 1.06)	435 per 1,000	**22 fewer per 1,000**<br>(from 70 fewer to 26 more)
**PONV**											
488<br>(6 RCTs)	serious[a]	not serious	not serious	serious[c]	none	⊕⊕◯◯<br>Low	83/245<br>(33.9%)	75/243<br>(30.9%)	**RR 0.95**<br>(0.69 to 1.31)	339 per 1,000	**17 fewer per 1,000**<br>(from 105 fewer to 105 more)
**Quality of recovery**											
446<br>(4 RCTs)	not serious	serious[b]	not serious	serious[c]	none	⊕⊕◯◯<br>Low	224	222	-	The mean quality of recovery was **0**	MD **0.22 higher**<br>(7.52 lower to 7.96 higher)
**Hospital stay**											
290<br>(3 RCTs)	serious[a]	not serious	not serious	serious[c]	none	⊕⊕◯◯<br>Low	146	144	-	The mean hospital stay was **0**	MD **1.11 lower**<br>(2.12 lower to 0.1 lower)

CI: confidence interval; **MD:** mean difference; **RR:** risk ratio

a. Some of the included trials are at unclear risk of bias.

b. Heterogeneity across trials was observed.

c.The included trials had wide 95% CIs.

Table S2. PRISMA 2020 checklist

Section and Topic	Item #	Checklist item	Location where item is reported

Section and Topic	Item #	Checklist item	Location where item is reported
**TITLE**			
Title	1	Identify the report as a systematic review.	1
**ABSTRACT**			
Abstract	2	See the PRISMA 2020 for Abstracts checklist.	2
**INTRODUCTION**			
Rationale	3	Describe the rationale for the review in the context of existing knowledge.	3-4
Objectives	4	Provide an explicit statement of the objective(s) or question(s) the review addresses.	4
**METHODS**			
Eligibility criteria	5	Specify the inclusion and exclusion criteria for the review and how studies were grouped for the syntheses.	5
Information sources	6	Specify all databases, registers, websites, organisations, reference lists and other sources searched or consulted to identify studies. Specify the date when each source was last searched or consulted.	4-5
Search strategy	7	Present the full search strategies for all databases, registers and websites, including any filters and limits used.	4
Selection process	8	Specify the methods used to decide whether a study met the inclusion criteria of the review, including how many reviewers screened each record and each report retrieved, whether they worked independently, and if applicable, details of automation tools used in the process.	4
Data collection process	9	Specify the methods used to collect data from reports, including how many reviewers collected data from each report, whether they worked independently, any processes for obtaining or confirming data from study investigators, and if applicable, details of automation tools used in the process.	4-5
Data items	10a	List and define all outcomes for which data were sought. Specify whether all results that were compatible with each outcome domain in each study were sought (e.g. for all measures, time points, analyses), and if not, the methods used to decide which results to collect.	6-7
	10b	List and define all other variables for which data were sought (e.g. participant and intervention characteristics, funding sources). Describe any assumptions made about any missing or unclear information.	6-7
Study risk of bias assessment	11	Specify the methods used to assess risk of bias in the included studies, including details of the tool(s) used, how many reviewers assessed each study and whether they worked independently, and if applicable, details of automation tools used in the process.	5-6
Effect measures	12	Specify for each outcome the effect measure(s) (e.g. risk ratio, mean difference) used in the synthesis or presentation of results.	6-7
Synthesis methods	13a	Describe the processes used to decide which studies were eligible for each synthesis (e.g. tabulating the study intervention characteristics and comparing against the planned groups for each synthesis (item #5)).	6-7
	13b	Describe any methods required to prepare the data for presentation or synthesis, such as handling of missing summary statistics, or data conversions.	6-7
	13c	Describe any methods used to tabulate or visually display results of individual studies and syntheses.	6-7
	13d	Describe any methods used to synthesize results and provide a rationale for the choice(s). If meta-analysis was performed, describe the model(s), method(s) to identify the presence and extent of statistical heterogeneity, and software package(s) used.	6-7
	13e	Describe any methods used to explore possible causes of heterogeneity among study results (e.g. subgroup analysis, meta-regression).	6-7
	13f	Describe any sensitivity analyses conducted to assess robustness of the synthesized results.	
Reporting bias assessment	14	Describe any methods used to assess risk of bias due to missing results in a synthesis (arising from reporting biases).	5-6
Certainty assessment	15	Describe any methods used to assess certainty (or confidence) in the body of evidence for an outcome.	5-6
**RESULTS**			

Section and Topic	Item #	Checklist item	Location where item is reported
Study selection	16a	Describe the results of the search and selection process, from the number of records identified in the search to the number of studies included in the review, ideally using a flow diagram.	8
	16b	Cite studies that might appear to meet the inclusion criteria, but which were excluded, and explain why they were excluded.	8
Study characteristics	17	Cite each included study and present its characteristics.	8
Risk of bias in studies	18	Present assessments of risk of bias for each included study.	8
Results of individual studies	19	For all outcomes, present, for each study: (a) summary statistics for each group (where appropriate) and (b) an effect estimate and its precision (e.g. confidence/credible interval), ideally using structured tables or plots.	9-10
Results of syntheses	20a	For each synthesis, briefly summarise the characteristics and risk of bias among contributing studies.	8
	20b	Present results of all statistical syntheses conducted. If meta-analysis was done, present for each the summary estimate and its precision (e.g. confidence/credible interval) and measures of statistical heterogeneity. If comparing groups, describe the direction of the effect.	8
	20c	Present results of all investigations of possible causes of heterogeneity among study results.	8
	20d	Present results of all sensitivity analyses conducted to assess the robustness of the synthesized results.	8
Reporting biases	21	Present assessments of risk of bias due to missing results (arising from reporting biases) for each synthesis assessed.	11
Certainty of evidence	22	Present assessments of certainty (or confidence) in the body of evidence for each outcome assessed.	10
**DISCUSSION**			
Discussion	23a	Provide a general interpretation of the results in the context of other evidence.	11-14
	23b	Discuss any limitations of the evidence included in the review.	14
	23c	Discuss any limitations of the review processes used.	14
	23d	Discuss implications of the results for practice, policy, and future research.	14
**OTHER INFORMATION**			
Registration and protocol	24a	Provide registration information for the review, including register name and registration number, or state that the review was not registered.	3
	24b	Indicate where the review protocol can be accessed, or state that a protocol was not prepared.	
	24c	Describe and explain any amendments to information provided at registration or in the protocol.	
Support	25	Describe sources of financial or non-financial support for the review, and the role of the funders or sponsors in the review.	14-15
Competing interests	26	Declare any competing interests of review authors.	15
Availability of data, code and other materials	27	Report which of the following are publicly available and where they can be found: template data collection forms; data extracted from included studies; data used for all analyses; analytic code; any other materials used in the review.	15

Study or Subgroup	Lidocaine Events	Lidocaine Total	Control Events	Control Total	Weight	Risk Ratio M-H, Random, 95% CI
**3 month**						
Grigoras 2012	2	17	9	19	6.4%	0.25 [0.06, 0.99]
Kendall 2018	18	75	13	75	18.8%	1.38 [0.73, 2.62]
Khan 2019	22	51	31	49	28.5%	0.68 [0.47, 1.00]
Kim 2017	7	42	14	42	14.4%	0.50 [0.22, 1.11]
Toner 2021	15	74	19	76	20.2%	0.81 [0.45, 1.47]
Xia 2022	5	41	13	41	11.7%	0.38 [0.15, 0.98]
**Subtotal (95% CI)**		**300**		**302**	**100.0%**	**0.68 [0.46, 0.99]**
Total events	69		99			
Heterogeneity: Tau² = 0.09; Chi² = 9.06, df = 5 (P = 0.11); I² = 45%						
Test for overall effect: Z = 2.01 (P = 0.04)						
**6 month**						
Kendall 2018	8	75	17	75	25.8%	0.47 [0.22, 1.02]
Terkawi 2015	4	40	8	40	14.5%	0.50 [0.16, 1.53]
Toner 2021	20	74	22	76	43.9%	0.93 [0.56, 1.56]
Xia 2022	4	41	11	41	15.8%	0.36 [0.13, 1.05]
**Subtotal (95% CI)**		**230**		**232**	**100.0%**	**0.62 [0.39, 0.98]**
Total events	36		58			
Heterogeneity: Tau² = 0.06; Chi² = 4.00, df = 3 (P = 0.26); I² = 25%						
Test for overall effect: Z = 2.06 (P = 0.04)						

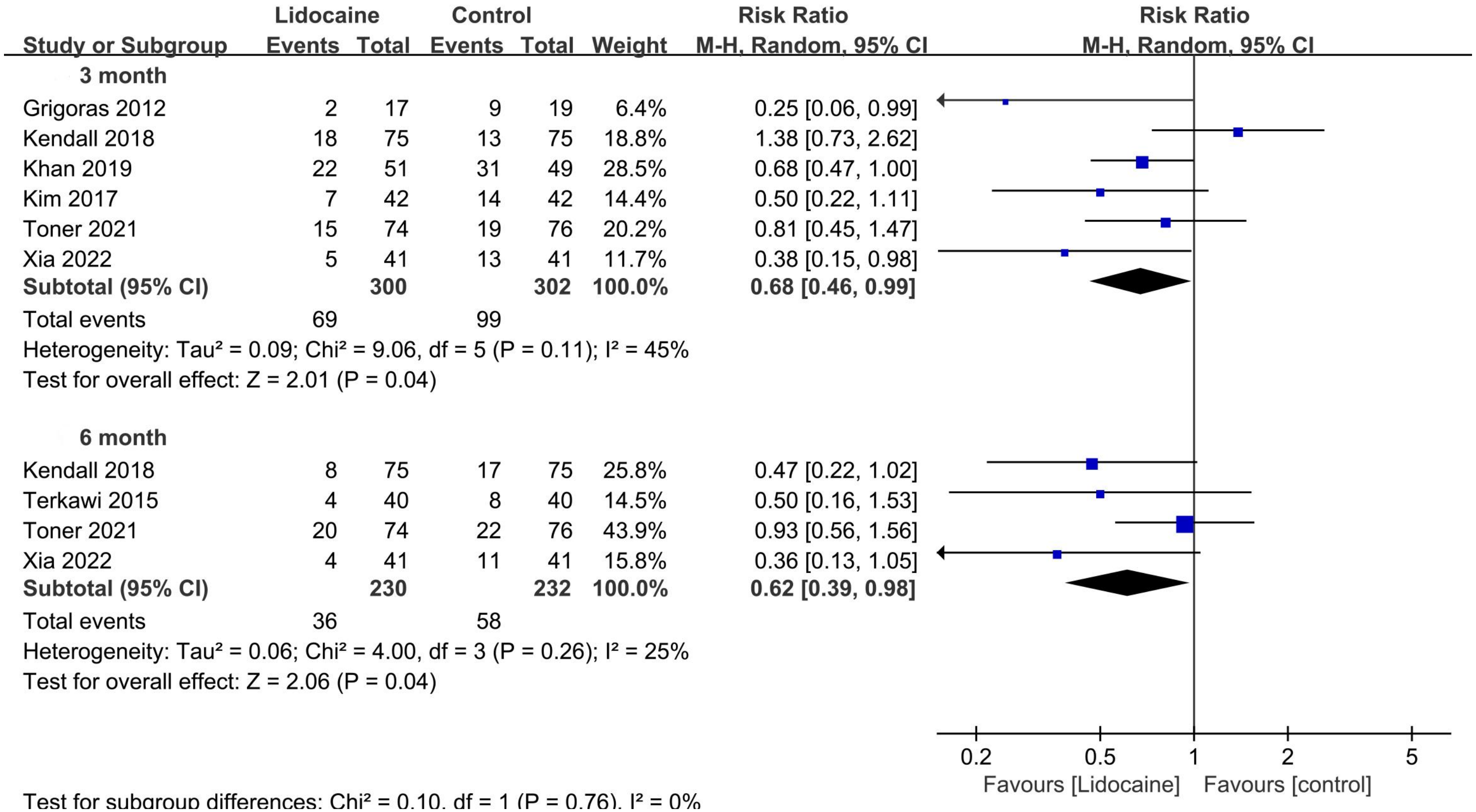

Test for subgroup differences: Chi² = 0.10, df = 1 (P = 0.76), I² = 0%

Figure S1 Forest plot for chronic post-surgical pain at three and six months follow-up after breast surgery

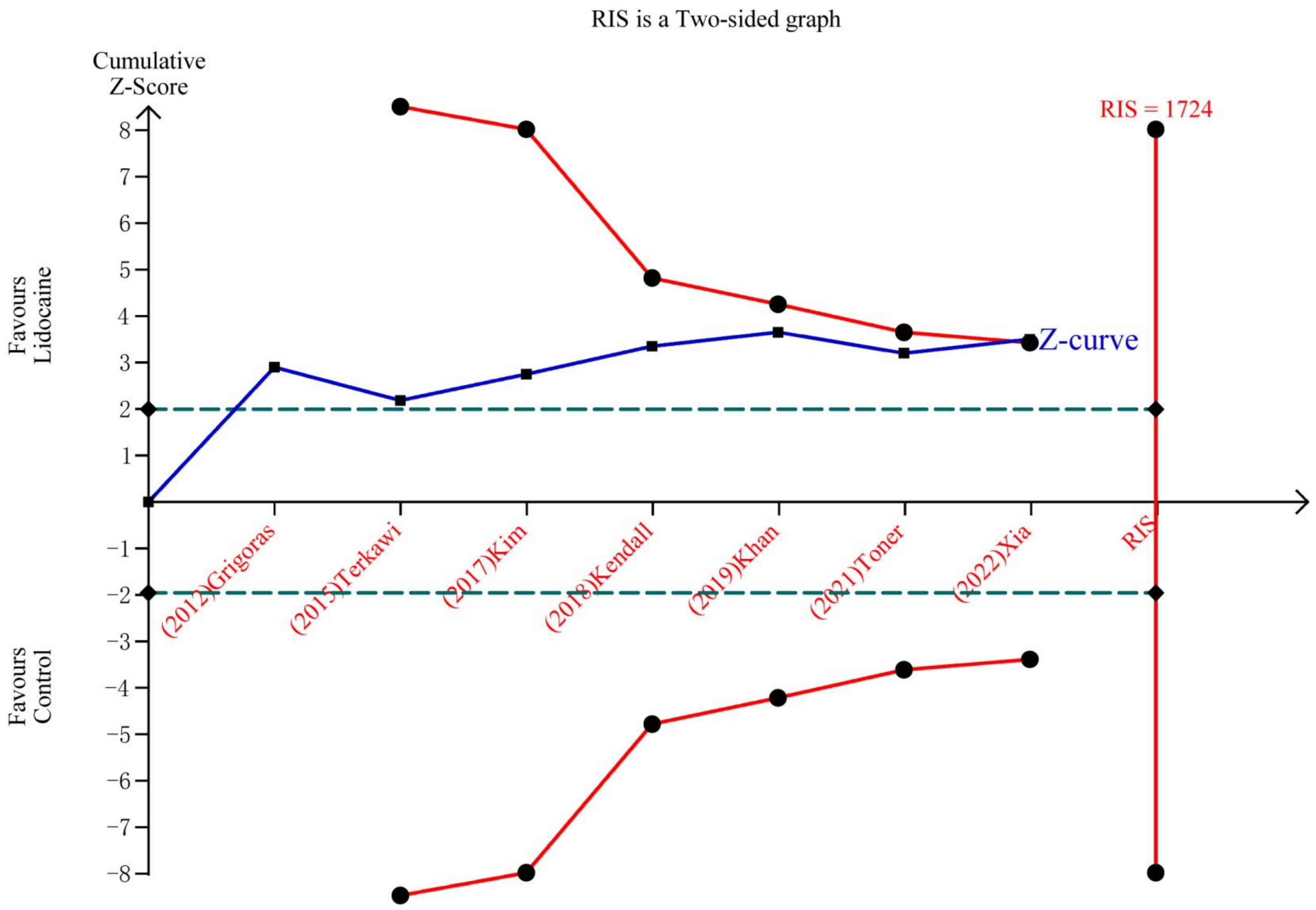

Figure S2 Trial sequential analysis for chronic post-surgical pain at the longest follow-up (scaled trial distance). Trial sequential analysis of seven trials (black filled squares) illustrating that the cumulative Z curve crossed the conventional boundary and the trial sequential monitoring boundary for benefit, establishing sufficient and conclusive evidence. A diversity-adjusted required information size of 1724 patients was calculated using α = 0.05 (two-sided), β= 0.20 (power of 80%), an anticipated relative risk reduction of 20%, and an event proportion of 32.75% in the control group.

Study or Subgroup	Lidocaine Mean	Lidocaine SD	Lidocaine Total	Control Mean	Control SD	Control Total	Weight	Mean Difference IV, Random, 95% CI
2017 Kim	313.7	116.4	42	498.4	150.2	42	78.7%	-184.70 [-242.17, -127.23]
2018 Kendall	881	413.33	74	1,061	591.11	74	9.6%	-180.00 [-344.34, -15.66]
2022 Wei	1,145	531.25	31	1,385.69	674.88	31	2.8%	-240.69 [-543.04, 61.66]
2022 Xia	1,022.4	454.07	41	1,225	331.85	41	8.8%	-202.60 [-374.75, -30.45]
**Total (95% CI)**			**188**			**188**	**100.0%**	**-187.41 [-238.41, -136.41]**

Heterogeneity: Tau² = 0.00; Chi² = 0.17, df = 3 (P = 0.98); I² = 0%

Test for overall effect: Z = 7.20 (P < 0.00001)

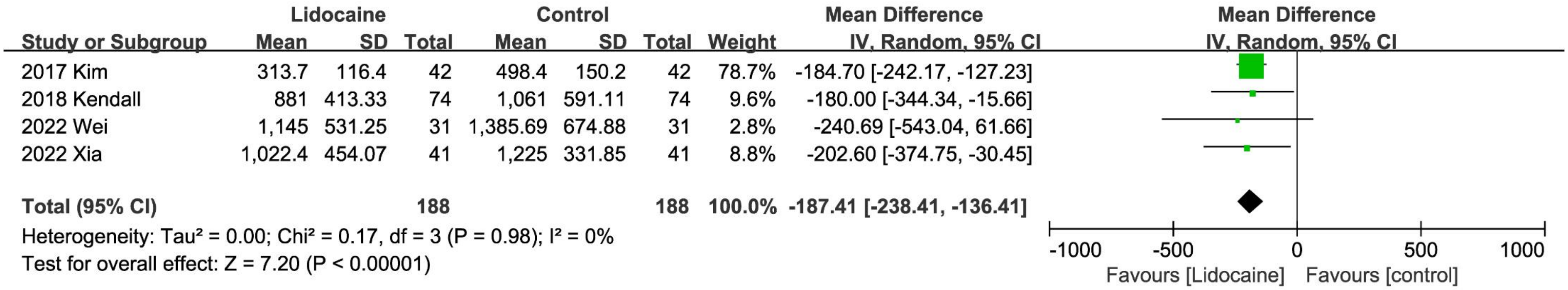

Figure S3 Forest plot for remifentanil (μg) consumption during breast surgery

	Lidocaine			Control				Mean Difference
**Study or Subgroup**	**Mean**	**SD**	**Total**	**Mean**	**SD**	**Total**	**Weight**	**IV, Random, 95% CI**
Grigoras 2012	2.1	5.1	17	4.2	10.3	19	0.2%	-2.10 [-7.33, 3.13]
Kendall 2018	34	21.48	75	39	26.67	75	0.1%	-5.00 [-12.75, 2.75]
Khan 2019	6.32	0.66	51	7.09	0.67	49	97.5%	-0.77 [-1.03, -0.51]
Kim 2017	4.6	4.5	42	5.3	4.1	42	2.0%	-0.70 [-2.54, 1.14]
Terkawi 2014	13	12.68	40	14.43	16.46	40	0.2%	-1.43 [-7.87, 5.01]
**Total (95% CI)**			**225**			**225**	**100.0%**	**-0.78 [-1.04, -0.52]**

Heterogeneity: Tau² = 0.00; Chi² = 1.44, df = 4 (P = 0.84); I² = 0%

Test for overall effect: Z = 5.92 (P < 0.00001)

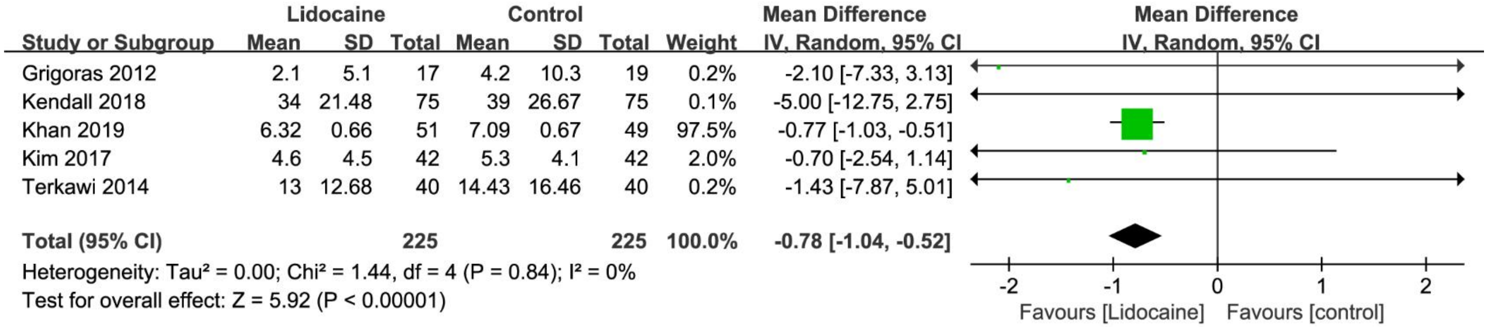

Figure S4 Forest plot for morphine(mg) consumption at 24h after breast surgery

Study or Subgroup	Lidocaine Events	Lidocaine Total	Control Events	Control Total	Weight	Risk Ratio M-H, Random, 95% CI
Choi 2012	4	30	1	30	0.3%	4.00 [0.47, 33.73]
Couceiro 2015	3	22	2	23	0.5%	1.57 [0.29, 8.51]
Grigoras 2012	13	17	14	19	9.2%	1.04 [0.71, 1.51]
Kim 2017	27	42	27	42	12.9%	1.00 [0.73, 1.38]
Toner 2021	61	74	68	76	76.9%	0.92 [0.81, 1.05]
Wei 2022	0	31	1	31	0.1%	0.33 [0.01, 7.88]
Xia 2022	0	41	1	41	0.1%	0.33 [0.01, 7.95]
**Total (95% CI)**		**257**		**262**	**100.0%**	**0.95 [0.84, 1.06]**
Total events	108		114			

Heterogeneity: Tau² = 0.00; Chi² = 3.75, df = 6 (P = 0.71); I² = 0%

Test for overall effect: Z = 0.97 (P = 0.33)

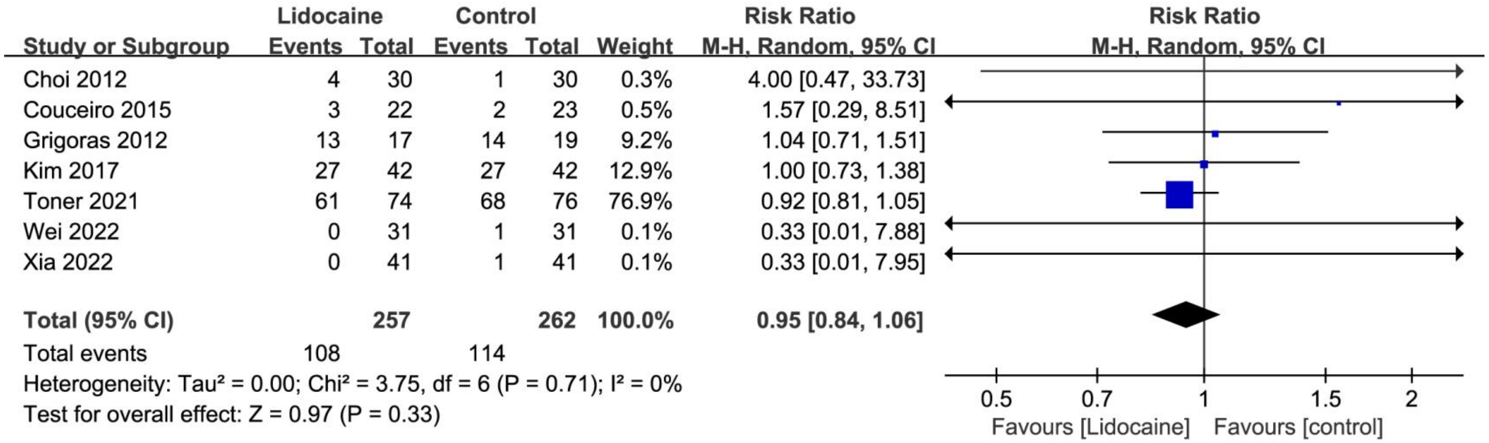

Figure S5 Forest plot for morphine consumption during surgery

Study or Subgroup	Lidocaine Events	Lidocaine Total	Control Events	Control Total	Weight	Risk Ratio M-H, Random, 95% CI
Choi 2012	22	30	16	30	31.1%	1.38 [0.92, 2.05]
Grigoras 2012	1	17	1	19	1.4%	1.12 [0.08, 16.52]
Khan 2019	11	51	16	49	17.0%	0.66 [0.34, 1.28]
Terkawi 2014	11	40	9	40	13.7%	1.22 [0.57, 2.62]
Toner 2021	28	74	36	76	32.8%	0.80 [0.55, 1.16]
Wei 2022	2	31	5	31	4.0%	0.40 [0.08, 1.91]
**Total (95% CI)**		**243**		**245**	**100.0%**	**0.95 [0.69, 1.31]**
Total events	75		83			

Heterogeneity: Tau² = 0.05; Chi² = 7.17, df = 5 (P = 0.21); I² = 30%

Test for overall effect: Z = 0.32 (P = 0.75)

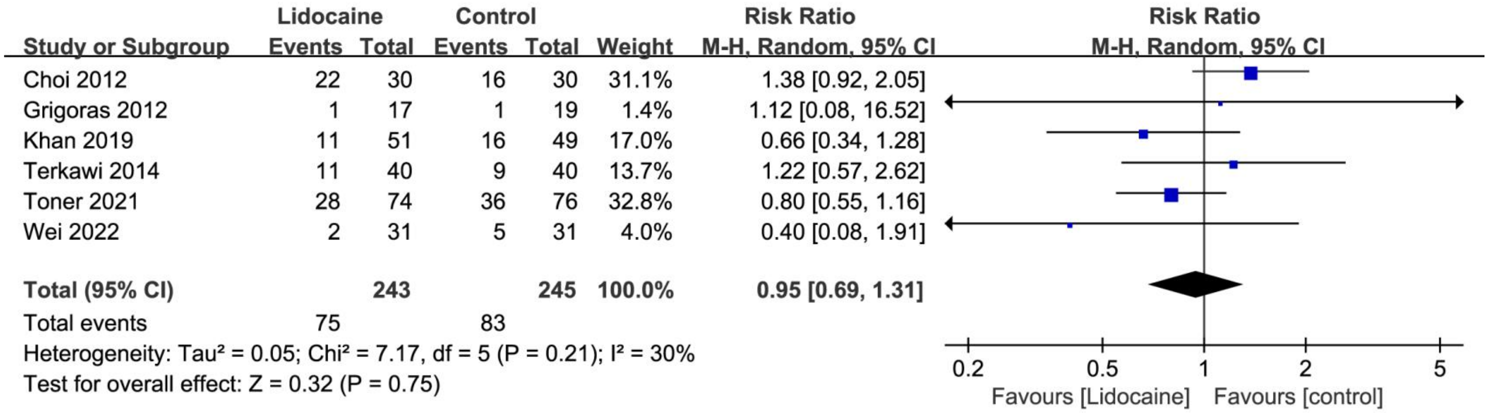

Figure S6 Forest plot for PONV within 24h breast surgery

Study or Subgroup	Lidocaine Events	Lidocaine Total	Control Events	Control Total	Weight	Risk Ratio M-H, Random, 95% CI
Choi 2012	4	30	1	30	0.3%	4.00 [0.47, 33.73]
Couceiro 2015	3	22	2	23	0.5%	1.57 [0.29, 8.51]
Grigoras 2012	13	17	14	19	9.2%	1.04 [0.71, 1.51]
Kim 2017	27	42	27	42	12.9%	1.00 [0.73, 1.38]
Toner 2021	61	74	68	76	76.9%	0.92 [0.81, 1.05]
Wei 2022	0	31	1	31	0.1%	0.33 [0.01, 7.88]
Xia 2022	0	41	1	41	0.1%	0.33 [0.01, 7.95]
**Total (95% CI)**		**257**		**262**	**100.0%**	**0.95 [0.84, 1.06]**
Total events	108		114			

Heterogeneity: $Tau^2 = 0.00$; $Chi^2 = 3.75$, df = 6 (P = 0.71); $I^2 = 0\%$

Test for overall effect: Z = 0.97 (P = 0.33)

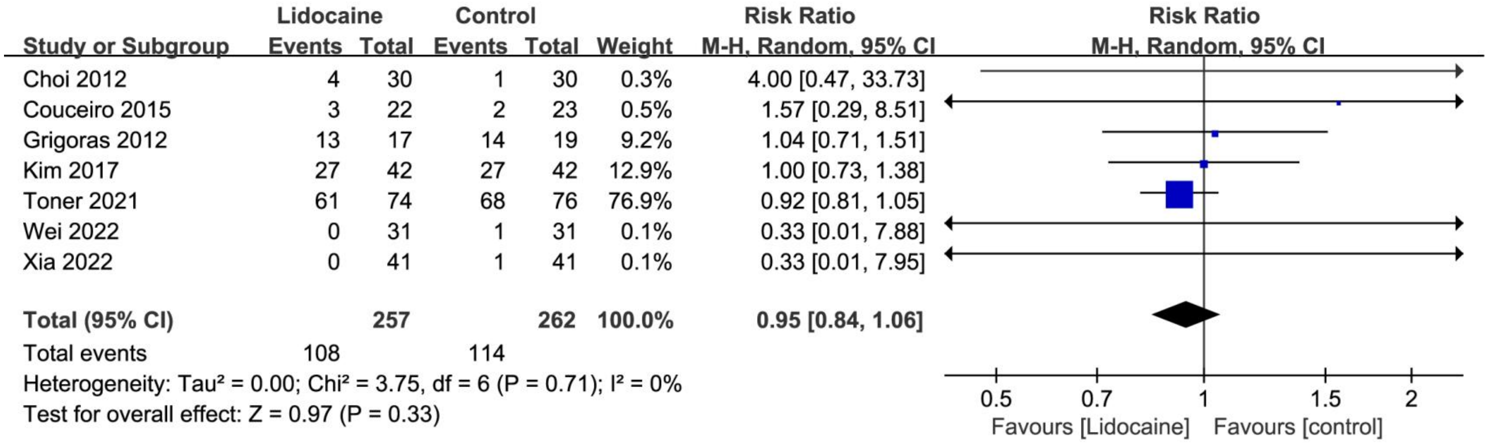

Figure S7 Forest plot showing rescue analgesic administration within 24h surgery

	Lidocaine			Control				Mean Difference
Study or Subgroup	Mean	SD	Total	Mean	SD	Total	Weight	IV, Random, 95% CI
Choi 2012	264	40.8	30	268.8	112.8	30	0.1%	-4.80 [-47.72, 38.12]
Terkawi 2014	30	8.89	40	32	4.44	40	10.8%	-2.00 [-5.08, 1.08]
Toner 2021	23	3.7	74	24	2.96	76	89.1%	-1.00 [-2.07, 0.07]
**Total (95% CI)**			**144**			**146**	**100.0%**	**-1.11 [-2.12, -0.10]**

Heterogeneity: $Tau^2 = 0.00$; $Chi^2 = 0.39$, df = 2 (P = 0.82); $I^2 = 0\%$

Test for overall effect: Z = 2.15 (P = 0.03)

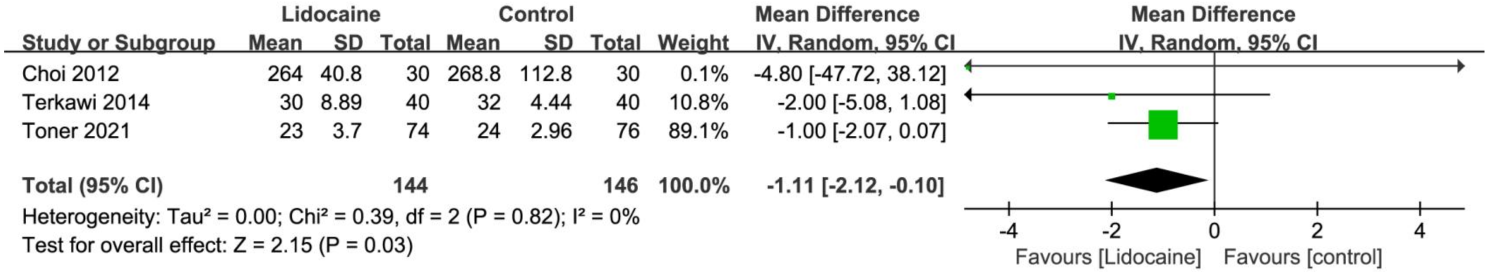

Figure S8 Forest plot for length of hospital stay (in hours) after surgery

Study or Subgroup	Lidocaine Mean	Lidocaine SD	Lidocaine Total	Control Mean	Control SD	Control Total	Weight	Mean Difference IV, Random, 95% CI
**2.7.1 QOR 40**								
Kendall 2018	158	22.96	75	169	18.52	75	24.3%	-11.00 [-17.68, -4.32]
Kim 2017	179.3	6.8	42	173.9	8.4	42	28.2%	5.40 [2.13, 8.67]
**Subtotal (95% CI)**			**117**			**117**	**52.5%**	**-2.53 [-18.59, 13.53]**
Heterogeneity: $Tau^2 = 127.29$; $Chi^2 = 18.70$, df = 1 ($P < 0.0001$); $I^2 = 95\%$								
Test for overall effect: Z = 0.31 (P = 0.76)								
**2.7.2 QOR 15**								
Toner 2021	132	14.81	74	135	8.89	76	27.6%	-3.00 [-6.92, 0.92]
Wei 2022	128.5	20.25	31	117.5	19.5	31	20.0%	11.00 [1.10, 20.90]
**Subtotal (95% CI)**			**105**			**107**	**47.5%**	**3.23 [-10.40, 16.87]**
Heterogeneity: $Tau^2 = 83.25$; $Chi^2 = 6.64$, df = 1 ($P = 0.010$); $I^2 = 85\%$								
Test for overall effect: Z = 0.46 (P = 0.64)								
**Total (95% CI)**			**222**			**224**	**100.0%**	**0.22 [-7.52, 7.96]**
Heterogeneity: $Tau^2 = 52.61$; $Chi^2 = 27.29$, df = 3 ($P < 0.00001$); $I^2 = 89\%$								
Test for overall effect: Z = 0.06 (P = 0.96)								
Test for subgroup differences: $Chi^2 = 0.29$, df = 1 ($P = 0.59$), $I^2 = 0\%$								

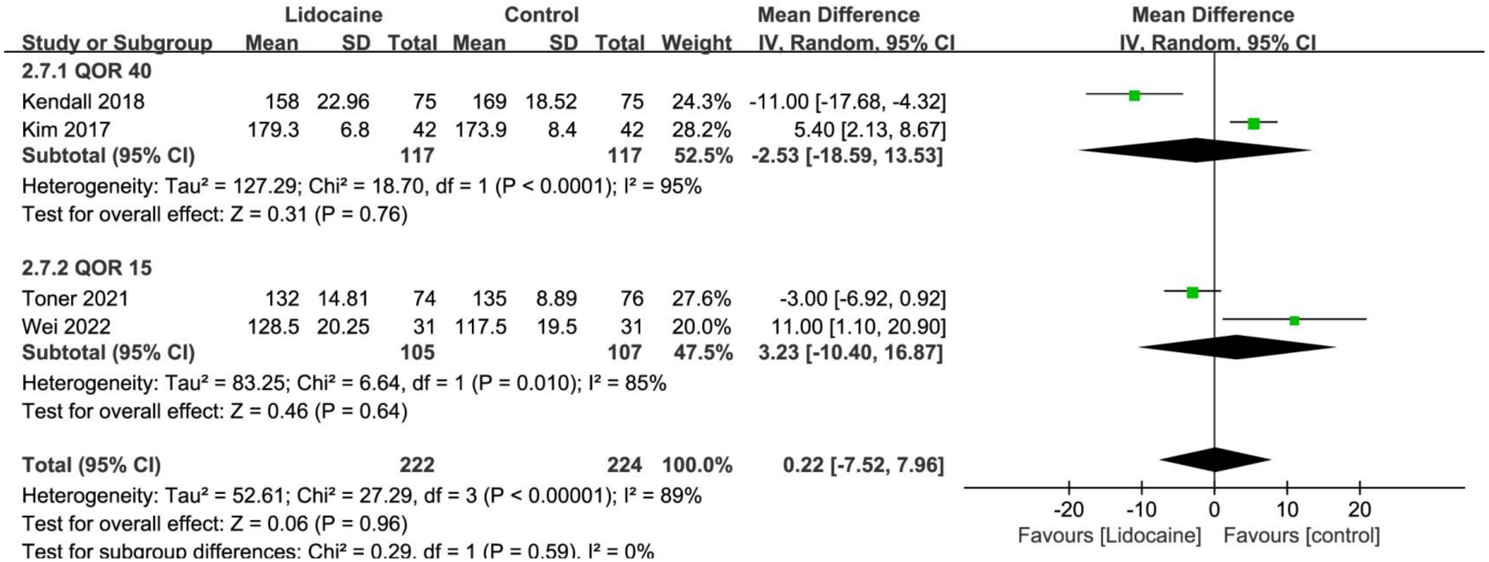

Figure S9 Forest plot showing quality of postoperative recovery within 24h after surgery

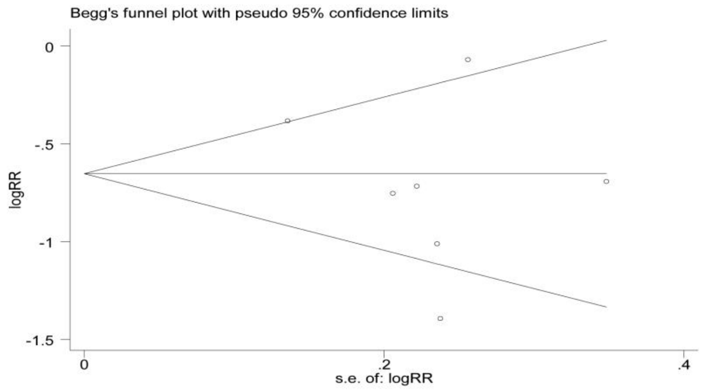

Tests for Publication Bias

Begg's Test

adj. Kendall's Score (P-Q) = -3
Std. Dev. of Score = 6.66
Number of Studies = 7
z = -0.45
Pr > |z| = 0.652
z = 0.30 (continuity corrected)
Pr > |z| = 0.764 (continuity corrected)

Egger's test

Std_Eff	Coef.	Std. Err.	t	P>|t|	[95% Conf. Interval]	
slope	-.1529349	.566483	-0.27	0.798	-1.609126	1.303256
bias	-2.46422	2.688175	-0.92	0.401	-9.374395	4.445954

Figure S10 Begg's test for evaluating publication bias. RR, relative risks
